# TRENDS IN FRAILTY INCIDENCE AND THE IMPACT OF LIFESTYLE AMONG US ADULTS AGED ≥50 YEARS FROM 2004 TO 2020

**DOI:** 10.1093/geroni/igad104.3045

**Published:** 2023-12-21

**Authors:** Hui-wen Xu, Yu-Ming Chen, Zhou Yang, Bei-bei Xu

**Affiliations:** Peking University, Beijing, Beijing, China (People’s Republic); Peking University, Beijing, Beijing, China (People’s Republic); Peking University, Beijing, Beijing, China (People’s Republic); Peking University, Beijing, Beijing, China (People’s Republic)

## Abstract

Few studies have estimated frailty incidence. The contribution of lifestyle to the incidence of frailty may change over time. It is necessary to quantify the impact of lifestyle on frailty incidence over time. A total of 25,639 subjects aged ≥50 years from the Health and Retirement Study (HRS) 2004-2020 were divided into seven consecutive and overlapping cohorts. Frailty was measured using the Paulson–Lichtenberg Frailty Index. Lifestyle included smoking status, alcohol consumption, physical activity, and sleep problems. The trend of frailty incidence rates was estimated by generalized estimating equation (GEE) models with a natural cubic spline. Population attributable fractions (PAFs) were calculated using hazard ratios from Cox models. Lifestyle-attributable frailty incidence rates were estimated by multiplying PAFs by the overall incidence rate of frailty. The incidence (per 1,000 person-years) of frailty showed a general trend of first decreasing and then increasing, with a peak occurrence of 22.7 (95% CI: 21.0, 24.5) in 2010–2014. Almost 20% of frailty incidence rates were attributed to current smoking, physical inactivity, or sleep problems. The incidence of frailty showed a fluctuating but decreasing trend among middle-aged and older adults in the United States from 2004 to 2020, with a peak occurrence after the Great Recession of 2007–2009. Smoking, physical inactivity, and sleep problems may have contributed importantly to the incidence of frailty. Effective and early frailty prevention strategies should be developed to reduce smoking, physical inactivity, and sleep problems, especially in the context of the global economic crisis caused by the COVID-19 epidemic.

